# Outcomes of decompression for lumbar spinal canal stenosis based upon preoperative radiographic severity

**DOI:** 10.1186/1749-799X-2-3

**Published:** 2007-03-08

**Authors:** Bradley K Weiner, Nilesh M Patel, Matthew A Walker

**Affiliations:** 1 Division of Spinal Surgery, Department of Orthopaedics, The Methodist Hospital/Texas Medical Center, Houston, Texas USA; 2 Department of Orthpaedics, Summa Health Systems, Akron, Ohio USA

## Abstract

**Background:**

The relationship between severity of preoperative radiographic findings and surgical outcomes following decompression for lumbar degenerative spinal canal stenosis is unclear. Our aim in this paper was to gain insight into this relationship. We determined pre-operative radiographic severity on MRI scans using strict methodological controls and correlated such severity with post-operative outcomes using prospectively collected data.

**Methods:**

Twenty-seven consecutive patients undergoing decompression for isolated degenerative spinal canal stenosis at L4-L5 were included. We measured cross-sectional area on MRI using the technique of Hamanishi. We categorized the severity of stenosis using Laurencin and Lipson's 'Stenosis Ratio'. We determined pre-operative status (prospectively) and post-operative outcomes using Weiner and Fraser's 'Neurogenic Claudication Outcome Score'. We determined patient satisfaction using standardized questionnaires. Each of these is a validated measure. Formal statistical evaluation was undertaken.

**Results:**

*No *patients (0 of 14) with a greater than 50% reduction in cross-sectional area on pre-operative MRI had unsatisfactory outcomes. In contrast, outcomes for patients with less than or equal to 50% reduction in cross-sectional area had unsatifactory outcomes in 6 of 13 cases, with all but one negative outcome having a cross-sectional area reduction between 32% and 47%.

**Conclusion:**

The findings suggest that there appears to be a relationship between severity of stenosis and outcomes of decompressive surgery such that patients with a greater than 50% reduction in cross sectional area are more likely to have a successful outcome.

## Background

The prognosis for a satisfactory outcome following lumbar decompressive surgery for degenerative spinal canal stenosis depends upon several factors such as comorbid diabetes, peripheral vascular disease, and cardiopulmonary insufficiency which are known to have a negative impact [1]. Another factor, the degree of preoperative spinal canal stenosis, may also be of prognostic significance. However, the current literature is unclear as to its importance. Separate studies by Herno[2], Airaksinen[3], K-E. Johnson[4], and B. Johnson[5] all found a correlation between the severity of stenosis and the surgical outcome. In contrast, independent studies by Surin[6] and Paine[7] showed patients with milder stenosis did better post-operatively. Finally, Amundsen[8] and Mariconda[9] found no correlation between severity of stenosis and outcomes. These variable findings may have several potential sources. Different radiographic techniques to measure and categorize the severity of stenosis have been used. In most studies, outcomes were assessed retrospectively and often without validated outcome measures. The patients had variable co-morbidities. The patients evaluated presented with different symptom complexes (neurogenic claudication versus radiculopathy versus isolated low back pain) and/or multiple levels of anatomic involvement (+/- degenerative instability) which may have had an affect on surgical outcomes independent of the degree of stenosis.

The question is an important one which deserves re-evaluation. Faced daily with patients who have severe stenosis, it is unclear whether such severity makes a difference in how they will respond to surgical intervention. On one hand, animal model research has demonstrated that prolonged neurologic compression results in irreversible damage including intraneural fibrosis at the root level and plastic changes in nociceptive transmission at the cord level [10-13]. This suggests that prolonged, severe compression may correlate with poor outcomes. On the other hand is the anecdotal and logical experience of most surgeons---the greater the pre-operative compression, the greater anatomic difference the surgical decompression makes, and the results are likely to follow.

Accordingly, the aim of this study was to better delineate the relationship between pre-operative radiographic severity and post-operative outcomes by paying strict attention to methodological controls to limit confounding factors outside of degree of compression.

## Methods

### Patient Population

Twenty-seven consecutive patients undergoing surgery between January 1998 and January 2000 who satisfied the following criteria were included:

*1. They had isolated spinal canal stenosis at the L4-L5 level and underwent a single level decompression*. The study was limited to single level cases to avoid quantification problems associated with multi-level stenosis with varying degrees of severity.

*2. The stenosis was degenerative; *defined as isolated to one segment (L-4-L5) and compression most significantly due to disc bulge and hypertrophic/buckled ligamentum flavum (the classic 'napkin ring' configuration). This afforded the use an internal control to determine the 'stenosis ratio'[14].

*3. They had neurogenic claudication with no radicular component*. The clinical syndromes and surgical outcomes appear to differ between stenosis patients with neurogenic claudication versus those with acute monoradiculopathies. The syndrome of neurogenic claudication is characterized in Table 1.

**Table 1:** Neurogenic Claudication

All patients in this study had 'classic' neurogenic claudication defined as:
1. Bilateral posterior thigh and, often, calf discomfort characterized by pain, parasthesias, tiredness, and heaviness.
2. Brought on by walking (usually < a city block) and standing (usually < five minutes).
3. Relieved by sitting or lying down.
4. Positive MRI demonstrating canal stenosis.
5. Absence of significant vascular impairment to the lower extremities, absence of peripheral neuropathy, absence of severe DJD of hips, and absence of cardiopulmonary insufficiency.

*4. They had MRI's with a minimum 1.5 Telsa and axial images obtained at right angles to the anatomic segment measured *to facilitate accurate measurement of cross-sectional areas,

*5. They had pre-operatively filled out the Neurogenic Claudication Outcome Score(NCOS) and were available for a minimum twenty month post-op follow-up*. The NCOS[15] has been previously validated as an outcome measure in stenosis.

*6. They did not have comordities including diabetes, peripheral vascular disease, cardiopulmonary insufficiency, severe hip disease, or a degenerative spondylolisthesis*.

### Outcome Measure

**Figure 1:**
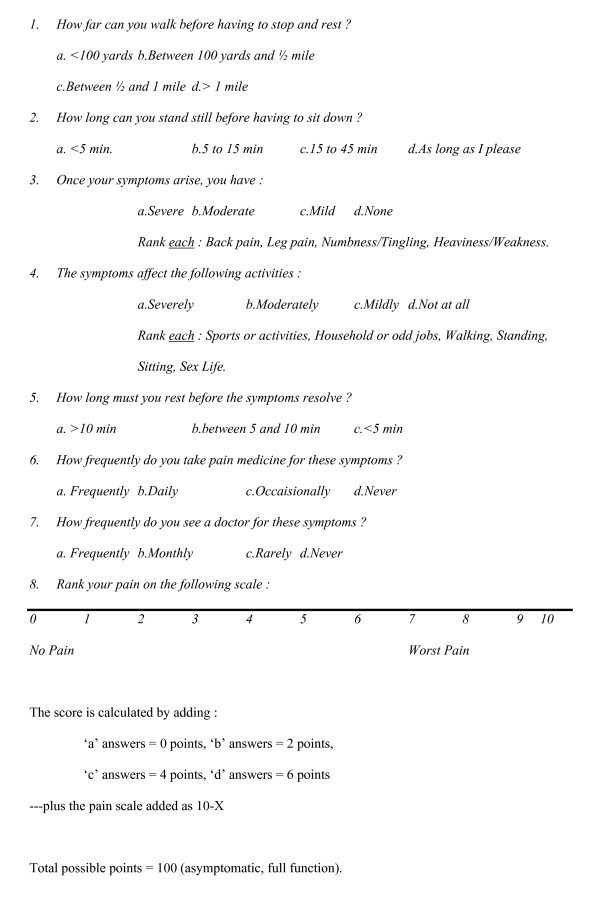
Neurogenic Claudication Outcome Score.

The NCOS questionnaire is shown in Figure 1. We also assessed patient satisfaction using a standardized form as shown in Table 2.

**Table 2:** Satisfaction Measures

1. Overall, how successful has your operation been?
**a**. Very successful, complete relief
**b**. Fairly successful, a good deal of relief
**c**. Not very successful, only a little relief
**d**. Failure, no relief
**e**. Worse than before
If you had a friend with the same trouble you had, would you recommend the operation? Yes/No
'Satisfaction' requires **a **or **b **and **Yes **to the above questions.

### Radiographic Measure

**Figure 2:**
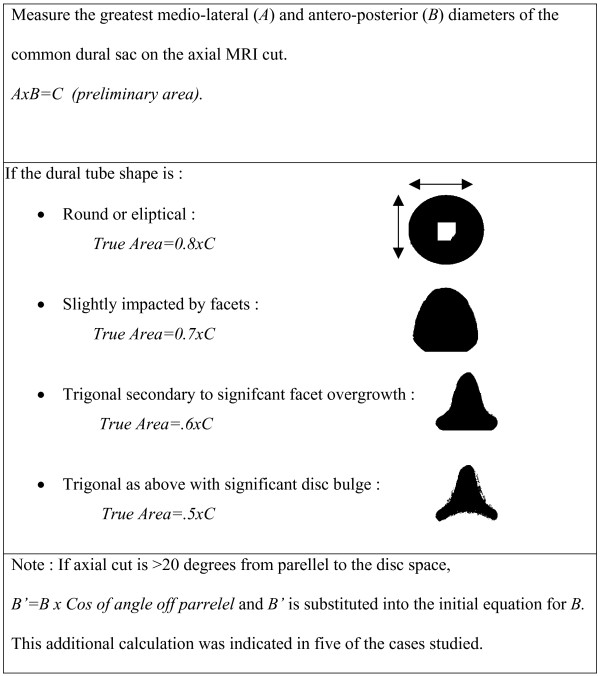
Hamanishi Technique to Measure Cross-sectional Area on Axial MRI.

We used the technique described by Hamanishi[16] (Figure 2) to determine the cross sectional area at the level demonstrating the most severe stenosis (using the method) and at the pedicle level uninvolved by stenosis. The 'stenosis ratio', as described by Lurencin and Lipson(14), was then used to determine the severity of stenosis. This ratio is the cross-sectional area of the canal at the axial MRI image with greatest neurologic compression (in these cases, L4-L5 disc level) over the cross-sectional area at the pedicle level above (in these cases, the pedicle level of L4). Two independent surgeons performed the measurements and calculations. They were blinded to each other's measurements as well as the patient's outcomes.

### Statistics

We used the Student *t-test *to measure significance of stenosis ratio versus change in NCOS. We used the *chi-square test *to measure significance of stenosis ratio versus patient satisfaction. To test the correlation between the two independent readers of MRI's, we used the *Pearson correlation coefficient*.

### Surgery

All patients underwent a lumbar decompression at L4-L5 using a previously described technique [17] performed by a single surgeon who was not directly involved in the study. The technique is one of microdecompression addressing the unilateral side via laminotomy/partial medical facetectomy and the contralateral side by angulation of the microscope and working under the midline structures to perform similar decompression. It is a minimally invasive technique affording outcomes commensurate with open laminectomy in propspective outcome studies[17].

## Results

Twenty-seven patients were studied. Their demographic data, area measurements, stenosis ratios, and NCOS scores are shown in Tables 3 and 4. The average age was 62 with a range from 37–83. There were 18 females and nine males. The average follow-up was 29 months with a range of 20 to 48 months. The average pre-operative NCOS was 4, and the average post-operative NCOS was 67. The duration of claudicant symptoms prior to surgery ranged between 6 months and 72 months and averaged 20 months. The interobserver correlation coefficient for measurement of area was 0.91 with p < .001. The three cases where the areas were measured with significant difference between observers was resolved by the senior author.

**Table 3:** Patient Data

Age	F/U (Mon)	Stenotic Area (mm2)	Pedicle Area (mm2)	Stenosis Ratio	Pre-op NCOS	Post-op NCOS	Change NCOS	Satisfaction
74	20	28	143	.20	7	67	60	Satisfied
77	22	24	109	.22	3	56	53	Satisfied
52	25	38	166	.23	2	51	49	Satisfied
52	22	50	205	.24	0	78	78	Satisfied
72	49	29	116	.25	7	98	91	Satisfied
83	39	64	208	.31	0	60	60	Satisfied
83	43	67	208	.32	9	100	91	Satisfied
77	27	69	193	.36	2	60	58	Satisfied
73	24	39	108	.36	6	80	74	Satisfied
60	29	81	221	.37	4	90	86	Satisfied
65	27	92	224	.41	5	98	93	Satisfied
74	22	66	151	.43	6	100	94	Satisfied
50	29	51	115	.44	1	75	74	Satisfied
58	24	67	150	.45	0	94	94	Satisfied
81	21	77	154	.50	7	25	18	Unsatisfied
67	20	79	159	.50	1	86	85	Satisfied
60	40	119	238	.50	2	45	43	Satisfied
59	26	59	112	.53	5	80	75	Satisfied
72	36	92	173	.53	7	52	45	Unsatisfied
59	36	49	88	.55	5	20	15	Unsatisfied
54	29	41	73	.56	5	45	40	Unsatisfied
54	20	95	144	.66	2	18	16	Unsatisfied
66	28	189	278	.68	4	33	29	Unsatisfied
76	48	141	203	.69	5	83	78	Satisfied
57	35	32	42	.77	1	70	69	Satisfied
37	19	114	146	.78	13	92	79	Satisfied
49	36	84	97	.87	2	51	49	Satisfied

**Table 4:** Outcomes Based on MRI Severity

**Stenosis Ratio< .5**		**Stenosis Ratio> or = .5**
*Greater than 50% reduction in cross-sectional area*		*Less than or equal to 50% reduction in cross-sectional area*
**Number**	**14**	**13**
**Satisfied**	14*	**7**
**Unsatisfied**	**0**	**6**
**Average** **Change in NCOS**	75*	**49**
• *Statistically Significant*		

As can be seen from the data, *no patients with greater than 50% reduction in cross-sectional area had unsatisfactory outcomes*, whereas those with less than or equal to 50% reduction in cross-sectional area had unsatisfactory outcomes in 6 of 13 cases---suggesting a potential threshold effect. That is, for cases with less than or equal to 50% reduction in cross-sectional area, greater variability in outcomes (greater likelihood of unsatisfactory outcome) can be anticipated. Cases with unsatisfactory outcomes, however, were clustered between a 32% and 47% reduction in cross-sectional area and, accordinlgy, the relationship between severity of stenosis and outcome does *not *appear to be linear. There was no statistical difference between cases with satisfactory outcomes and those with unsatisfactory outcomes in regards to duration of symptoms but power may be insufficient.

For the fourteen patients with greater than 50% reduction in cross-sectional area, NCOS improved an average of 75 points (range 52 to 94 points) and 100% were satisfied with the outcome. Of the thirteen with less than or equal to 50% reduction in cross-sectional area, the NCOS improved an average of 49 points (range 16 to 85 points) and only 50% were satisfied with the outcome. These findings were statistically significant at p < 0.05. It is of note that the starting point of the two groups was quite similar; there does not appear to be a ceiling effect.

## Discussion

Several animal models have demonstrated that rapid application of severe, prolonged compression of nerve roots may result in intraneural fibrosis which, despite decompressive intervention, may be irreversible [10-13,18]. These models mimic severe traumatic disc herniations and fractures and their associated syndromes. In such instances, the severity of neurolgic compression and the duration of compression likely relate directly to inferior neurologic outcomes.

Degenerative spinal canal stenosis with neurogenic claudication, however, is physiologically distinct from these more acute types of neurologic compression. The slowly progressive compression appears to afford the roots time to physiologically adjust to the changing situation such that many patients with severe narrowing of the spinal canal remain asymptomatic. There is, however, a subgroup of patients with milder degrees of stenosis who clearly present with neurogenic claudication suggesting that factors *intrinsic *to the roots may diminish their ability to physiologically adjust to compression. Based upon the findings in the current study, one might hypothesize that those patients who present with more severe spinal canal stenosis have roots which physiologically are better able to withstand progressive neurologic compression (hence they present later in the process) and, acordingly, these roots are better able to recover physiologically following decompression. By the same token, patients presenting with neurogenic claudication with milder amounts of stenosis may have roots which are physiologically more susceptible and such roots may be less likely to recover following decompression---the development of neurogenic claudication in these patients may relate more to poor physiologic reserves than the actual severity of compression.

This also appears to be the case in patients with comorbidities (who were excluded from the current study) such as significant diabetes (especially with neuropathy), vascular disease, and cardiopulmonary insufficiency. Physiologic changes in baseline nerve root nutrition may inhibit recovery following decompression.

In summary, we have found that patients with a greater than 50% reduction in cross-sectional area using the described technique of measurement appear to have a better (more predictably positive) outcome following decompressive surgery. Accordinlgy, those patients presenting with true neurogenic claudication but milder degrees of stenosis deserve greater attention. Checking EMG/NCT's to rule out neuropathy, checking non-invasive arterial studies to rule out vascular disease, re-reviewing hip x-rays and lateral flexion lumbar films to rule out degenerative joint disease or spondylolisthesis is appropriate. One might also consider attaining upright/weight-bearing MRI studies (as these become more readily available) which may provide better insight into the dynamic aspects of stenosis and may have prognostic importance. If these are negative, a realistic picture regarding potential outcomes of surgical intervention should be presented---acknowledging that the anticipated outcomes in this subgroup may be worse than those in whom more severe stenosis is present.

## Competing interests

None of the authors has any financial or non-financial competing interest in this study.

## Authors' contributions

NP and MW collected and statistically analyzed the data. BW conceived, designed, and wrote the paper. Each author read and approved the final manuscript.
